# Detection of multi-drug resistant *Escherichia coli* in the urban waterways of Milwaukee, WI

**DOI:** 10.3389/fmicb.2015.00336

**Published:** 2015-04-29

**Authors:** Anthony D. Kappell, Maxwell S. DeNies, Neha H. Ahuja, Nathan A. Ledeboer, Ryan J. Newton, Krassimira R. Hristova

**Affiliations:** ^1^Department of Biological Sciences, Marquette UniversityMilwaukee, WI, USA; ^2^Department of Pathology, Medical College of WisconsinMilwaukee, WI, USA; ^3^Dynacare Laboratories, MilwaukeeWI, USA; ^4^School of Freshwater Sciences, Great Lakes WATER Institute, University of Wisconsin–MilwaukeeMilwaukee, WI, USA

**Keywords:** antibiotic resistance genes, antibiotic resistance bacteria, Great Lakes, sediment, environment, *E. coli*

## Abstract

Urban waterways represent a natural reservoir of antibiotic resistance which may provide a source of transferable genetic elements to human commensal bacteria and pathogens. The objective of this study was to evaluate antibiotic resistance of *Escherichia coli* isolated from the urban waterways of Milwaukee, WI compared to those from Milwaukee sewage and a clinical setting in Milwaukee. Antibiotics covering 10 different families were utilized to determine the phenotypic antibiotic resistance for all 259 *E. coli* isolates. All obtained isolates were determined to be multi-drug resistant. The *E. coli* isolates were also screened for the presence of the genetic determinants of resistance including *ermB* (macrolide resistance), *tet*(M) (tetracycline resistance), and β-lactamases (*bla*_OXA_, *bla*_SHV_, and *bla*_PSE_). *E. coli* from urban waterways showed a greater incidence of antibiotic resistance to 8 of 17 antibiotics tested compared to human derived sources. These *E. coli* isolates also demonstrated a greater incidence of resistance to higher numbers of antibiotics compared to the human derived isolates. The urban waterways demonstrated a greater abundance of isolates with co-occurrence of antibiotic resistance than human derived sources. When screened for five different antibiotic resistance genes conferring macrolide, tetracycline, and β-lactam resistance, clinical *E. coli* isolates were more likely to harbor *ermB* and *bla*_OXA_ than isolates from urban waterway. These results indicate that Milwaukee’s urban waterways may select or allow for a greater incidence of multiple antibiotic resistance organisms and likely harbor a different antibiotic resistance gene pool than clinical sources. The implications of this study are significant to understanding the presence of resistance in urban freshwater environments by supporting the idea that sediment from urban waterways serves as a reservoir of antibiotic resistance.

## Introduction

The increasing number of multiple-antibiotic resistant pathogens has become a serious threat to human health (Centers for Disease Control and Prevention [CDC], 2013; Review on Antimicrobial Resistance [RAR], 2014; World Health Organization [WHO], 2014). Over the past two decades researchers have expanded their focus from the clinical settings to also include the natural environment as a reservoir of antibiotic resistance (Kümmerer, 2004; Baquero et al., 2008; Martinez et al., 2009; Allen et al., 2010; Galán et al., 2013; Michael et al., 2013; Wellington et al., 2013). The resistome of fecal bacteria from human and animal sources released into the environment impart antibiotic resistances genes to the non-resistant indigenous microorganisms (Aminov, 2011; Tacão et al., 2014). The subsequent transfer of antibiotic resistance genes from the indigenous microorganisms to human-associated bacteria may take place (Devirgiliis et al., 2011; Figueira et al., 2011). Simultaneously, positive selective pressure for antibiotic resistance genes in the environment may be stimulated by the presence of antibiotics or other contaminants (Silveira et al., 2014; Varela et al., 2014; Gao et al., 2015). These facts highlight the need to identify the potential sources of antibiotic resistant bacteria in environments used by human populations (Rosewarne et al., 2010; Gomez-Alvarez et al., 2012).

*Escherichia coli* are currently used by the Environmental Protection Agency (EPA, USA) as an indicator organism for fecal contamination and bacterial impairment for watersheds. *E. coli* is a natural member of intestinal microbiome of humans and other animals (reviewed in Harwood et al., 2014). The major sources of fecal contamination in various watersheds include human (Bernhard and Field, 2000), agricultural animals (Shanks et al., 2008), pets (Ervin et al., 2014), and wild animals (Somarelli et al., 2007; Guber et al., 2015), such as gulls (Alves et al., 2014; Araújo et al., 2014). The major source of fecal contamination of urban waterways of Milwaukee, WI was determined to be human (Newton et al., 2011, 2013).

Multi-drug resistant (MDR) *E. coli* and other *Enterobacteriaceae* isolates are characterized by non-susceptibility (or non-sensitivity) to at least one agent in three or more antibiotic categories (Magiorakos et al., 2012). Antibiotic resistance surveillance data show that *E. coli* has high resistance for older generation human and veterinary antibiotics including ampicillin, streptomycin, and tetracycline and the increasing resistance to newer antibiotics such as fluoroquinolones and cephalosporins (Tadesse et al., 2012).

*E. coli* had been recognized as a contributor to the dissemination of antibiotic resistance genes in natural environments (Henriques et al., 2006; Zhao and Dang, 2012; Alm et al., 2014; Alves et al., 2014). The gene encoding resistance to tetracycline class antibiotics, *tet*(M), which is predominately found on transposons within enterococci have also been found on plasmids within *E. coli* (Jurado-Rabadán et al., 2014), and possibly in *E. coli* from a natural river basin (Hu et al., 2008). The *ermB* gene encoding resistance to macrolides, lincosamides, and streptogramin have been identified on transposons and plasmids within or transferable to *E. coli* (Poyart et al., 1995; Poirel et al., 2011). The genes *bla*_OXA_, *bla*_SHV_, and *bla*_PSE_ are grouped in the most common types of β-lactamases belonging to *Enterobacteriaceae* (Bush and Jacoby, 2010).

Freshwater environments are recognized as reactors for the evolution and dissemination of antibiotic resistance (Alm et al., 2014; Czekalski et al., 2014; Marti et al., 2014; Vaz-Moreira et al., 2014), however, processes occurring in urban freshwater environments are less understood. In addition, the presence of antibiotic resistance *E. coli* in urban waterways represents a health issue in areas that are used for recreation activities.

The Milwaukee Harbor, an urbanized estuary, has a documented history of contamination from human activities. Located within the harbor is a wastewater treatment plant discharging the treated eﬄuent within the outer harbor into the Lake. High incidence of antibiotic resistance in *E. coli*, an indication of sewage contamination also was detected in storm water from the Menomonee River, which flows through the city of Milwaukee and into the Lake Michigan harbor (Salmore et al., 2006). Human fecal pollution is constant within the contributing rivers and the Milwaukee Harbor and increased contamination during heavy rain events has been reported (Newton et al., 2013). There is also evidence of high levels of personal care products and pharmaceuticals, including antibiotics present within the Milwaukee Harbor (Blair et al., 2013a). A study by LaPara et al. (2011) found that the presence of tetracycline resistance determinants *tet*(A), *tet*(X), and *tet*(W) in Lake Superior surface waters receiving Waste Water Treatment Plant (WWTP) eﬄuent near urban environments were correlated with the presence of fecal bacteria.

The objective of this study was to evaluate the abundance of multiple-antibiotic resistant bacteria present in the Milwaukee’s urban waterways compared to the human derived bacterial community from Milwaukee, WI. Since the urban waterways of Milwaukee have the potential for positive selection of antibiotic resistance due to history of antibiotics present (Blair et al., 2013a), we hypothesize that the *E. coli* isolated from urban waterways in Milwaukee maintain a similar or greater incidence of antibiotic resistance compared to the human derived *E. coli* isolates. The relationship of the resistances identified in the microbial community of the urban waterways and the human derived microbial community were explored in order to test this hypothesis. *E. coli* were isolated from sediment within the inner and outer Milwaukee harbor, human derived sewage, and from a clinical laboratory servicing Milwaukee. A broad range of antibiotics covering different families (β-lactams, aminoglycosides, tetracyclines, quinolones/fluoroquinolones, sulfonamides, dihydrofolate reductase inhibitors, UDP-*N*-acetylglucosamine enolpyruvyl transferase inhibitor, rifampicin, and chloramphenicol) were used to determine the resistance of the *E. coli* isolates. The presence of genetic determinates of resistance for *ermB* (macrolide resistance), *tet*(M), and β-lactamases (*bla*_OXA_, *bla*_SHV_, and *bla*_PSE_), were screened in the same *E. coli* isolates.

## Materials and Methods

### Study Site and Sample Collection

On March 22, 2012, four sediment grab samples at different locations were collected with a box corer from the Milwaukee Harbor Estuary. (A sampling map is shown in Supplementary Figure S1.) The Milwaukee Harbor Estuary is defined as the confluence of the Milwaukee, the Kinnickinnic, and the Menomonee Rivers into the Milwaukee Harbor. A subsample from each grab sample was collected in two sterile 50 mL conical centrifuge tubes and held at 4°C until filtering and plating. Strains isolated from the Kinnickinnic River and the junction of the Kinnickinnic River with the Milwaukee and Menomonee Rivers in the inner Milwaukee Harbor are referred to as the inner harbor isolates (*n* = 36). The strains referred to as the outer harbor isolates (*n* = 58) are further downstream of the junction of the rivers and were from sediment collected near the eﬄuent pipe of the Jones Island WWTP in the outer Milwaukee Harbor. Human derived sewage (*n* = 66) from the influent of the Jones Island WWTP was used for assessing microbial resistance of the human population. The influent WWTP water was placed in a pre-chilled 1 L bottle and kept at 4°C until filtering and plating. Disassociation of bacteria from sediment particles in the grab samples were performed as by Boehm et al. (2009). Briefly, grab samples (3 g) were diluted 1:10 in sterile buffered (pH 7.0) water and shook vigorously by hand for 2 min. The undiluted and serial diluted eluents from the sediment samples and the water samples from the influent of the WWTP were subjected to filtration through a 0.45 μm filter and the filter was placed on a modified membrane-thermotolerant *E. coli* Agar (modified mTEC) plate as in EPA Method 1603 (Environmental Protection Agency [EPA], 2002). As the EPA Method indicates the plates were initially incubated for 2 h at 35°C for recovery of injured cells followed by incubation at 44.5°C for 22 h. The colonies identified as *E. coli* based on pigmentation on the modified mTEC were recovered on Tryptic Soy Agar (TSA) media and incubated at 37°C for 18 h. *E. coli* strains of clinical consequence (*n* = 99) were obtained from Dynacare Laboratories (Milwaukee, WI, USA) on TSA slants. The clinical *E. coli* isolates were collected from various patient populations including outpatients and hospitalized patients from throughout the Milwaukee area and identity was confirmed by MALDI-TOF (Anderson et al., 2012; Buchan et al., 2012). All *E. coli* isolates were then grown in Tryptic Soy Broth (TSB) at 37°C for 18 h and stored at -20°C after the addition of glycerol to a final concentration of 10%. An additional 1 mL of culture was pelleted by centrifugation and stored at -20°C for DNA extraction.

### Antibiotic Susceptibility Testing

The *E. coli* isolates were tested for susceptibility to 17 antibiotics by a 96-well broth dilution method in Muller–Hinton broth utilizing three antibiotic concentrations based on the Clinical and Laboratory Standards Institute (Clinical and Laboratory Standards Institute [CLSI], 2011) guidelines. Four wells were used for each *E. coli* isolate consisting of three wells containing a serial half concentration dilution of the antibiotic and one well with no antibiotic as a positive control for growth. The 96-well plates containing 200 μL per well were incubated at 35°C for 18 h. The following antibiotics were used: ampicillin (AMP; Sigma, A0166; 8–32 μg mL^-1^), gentamicin (GEN; Sigma, G1264; 4–16 μg mL^-1^), streptomycin (STR; Sigma, S9137; 8–32 μg mL^-1^), neomycin (NEO; Sigma, N6386; 16–64 μg mL^-1^), tetracycline (TET; Sigma, T7660; 4–16 μg mL^-1^), ciprofloxacin (CPR; Sigma, 17850; 1–4 μg mL^-1^), chloramphenicol (CHL; Sigma, C1919; 8–32 μg mL^-1^), trimethoprim (TRM; Sigma, 92131; 8–32 μg mL^-1^), sulfamethoxazole (SFM; Sigma, S7507; 256–1024 μg mL^-1^), fosfomycin (FOS; Sigma, P5396; 64–256 μg mL^-1^), erythromycin (ERY; Sigma, E5389; 2–8 μg mL^-1^), aztreonam (AZT; Sigma, A6848; 4–16 μg mL^-1^), cefuroxime (CFX; Sigma, C4417; 8–32 μg mL^-1^), meropenem (MER; Sigma, M2574; 1–4 μg mL^-1^), moxifloxacin (MOX; Selleckchem, S1465; 1–4 μg mL^-1^), rifampicin (RIF; Sigma, R7382; 1–4 μg mL^-1^), and cefepime (CFP; US Pharmacopeial, 1097636; 8–32 μg mL^-1^). Isolates were classified as sensitive when growth was sequestered to the well without antibiotic and resistant when growth was observed in all four wells. Intermediate level of resistance was concluded when growth was observed in wells containing antibiotic concentrations diluted from the maximum. Where appropriate the classification of non-sensitive was used to denote any strain with any level of resistance to an antibiotic which includes Resistant or Intermediate levels determined by the Clinical and Laboratory Standards Institute (Clinical and Laboratory Standards Institute [CLSI], 2011).

### Antibiotic Resistance Gene Detection

*Escherichia coli* strains regardless of resistances were screened by real-time PCR to detect genes conferring resistance comprized of *tet(M)*, *ermB*, *bla*_OXA_, *bla*_SHV_, and *bla*_PSE_. Extractions of the total DNA of the isolates were performed with the Wizard Genomic DNA Purification Kit (Promega, A1120) on 1 mL of culture in TSB incubated at 37°C for 18 h. The PCR reactions were performed in a MyiQ or CFX Connect Real-Time PCR Detection Systems (Bio-rad, USA). Primers used are presented in Supplementary Table S1. The reaction mixture of 20 μL consisted of Standard *Taq* Buffer (10 mM Tris-HCl, 50 mM KCl, 1.5 mM MgCl_2_, pH 8.3; NEB, USA), 500 nM of each primer, 500 μM dNTPs, 0.5 U of *Taq* DNA Polymerase (NEB, USA), 0.1x SYBR Green I Nucleic Acid Gel Stain (Lonza, Cat#50513) and 25 ng of genomic DNA. PCR thermocycling conditions consisted of an initial denaturation step of 5 min at 95°C followed by 40 cycles of 95°C for 30 s, the gene specific annealing temperature (Supplementary Table S1) for 30 s, and 72°C for 30 s concluding with a melt curve analysis. Presence of the gene was determined by the cycle threshold and melt curve analysis of the real-time PCR results compared to a positive standard and no template controls. Amplifiable DNA was determined utilizing the 1369F and 1492R Bacterial 16S Primers (Suzuki et al., 2000).

Positive standards for PCR were generated by utilizing the PCR reaction mixture and conditions as used for screening without SYBR Green. DNA template for the PCR reactions to create standards was pooled DNA from clinical strains, not used in this study, with documented multiple resistances. PCR products at the expected product size determined by 2% agarose gel electrophoresis were excised from the gel, purified utilizing a QIAquick Gel Extraction Kit (Qiagen, USA), and cloned into the plasmid pUC19 (Yanisch-Perron et al., 1985). For cloning into pUC19, the plasmid was fully digested with SmaI endonuclease enzyme (NEB, USA) and the enzyme heat inactivated at 65°C for 30 min. The SmaI digested pUC19 was treated in a 50 μL reaction with Standard Taq Buffer, 0.5 U of Taq DNA Polymerase (NEB, USA), and 100 μM dTTP to generate 3′ T overhangs to aid in cloning the PCR products with 3′ A overhangs. The PCR products for the generation of standards were ligated into pUC19 utilizing T4 Ligase by manufacture’s recommended conditions (NEB, USA). Chemically competent TOP10 cells (Invitrogen, USA) were transformed with the ligation reaction and plated onto TSA augmented with 200 μg mL^-1^ ampicillin. Colony PCR was utilized to screen for isolates containing pUC19 with the insertions utilizing the M13 forward and reverse primers. Isolates demonstrating successful insertion of the PCR product into pUC19 were propagated and plasmids utilized for positive standards were isolated by I-Blue Mini Plasmid Kit (IBI, USA) and confirmed by Sanger sequencing.

Of the 295 positive amplification from the screening PCRs, 146 (49%) were purified utilizing the QIAquick PCR Purification Kit (Qiagen, USA) and sequenced utilizing their respective primers. All sequenced amplicons were confirmed to be the targeted genes (Results summarized in Supplementary Table S2).

### Statistical Analysis

Antibiotic resistance phenotypic profiles and gene presence was converted into numerical code. For each antibiotic: 1 signified susceptibility, 2 for intermediate resistance, and 3 represented resistance. The presence of a gene was signified as 1 and absence as 0. Principal component analysis (PCA) was performed using the package FactoMineR (Lê et al., 2008) from the open source statistical program R (R Core Team, 2012). Multivariate statistical analyses were performed using routines in the R package ‘vegan’ (Oksanen et al., 2013). Bray–Curtis similarities were calculated for antibiotic resistance levels and gene presence or absence using vegdist function in vegan package as input for multivariate ANOVA (MANOVA) analyses using the permutational alternative (Anderson, 2001) to standard parametric MANOVA provided by vegan’s ‘adonis’ function. Multiple pairwise tests were conducted using Tukey’s honestly significant differences (HSD) at the 5% family wise level of significance. The ‘glm’ function with the Poisson distribution and Analysis of Deviance for Generalized Liner Model utilizing the Chi-square test to determine significant differences between the antibiotic variable vectors and the source vectors.

Proportional *Z* test was utilized to identify significant differences between count data which is represented as percentages such as antibiotic resistances and presence of genes by location. Mann–Whitney *U* test was used for comparison of number of antibiotic resistance in the isolates from the locations.

## Results

### Antibiotic Resistance Profiles

**FIGURE 1:**
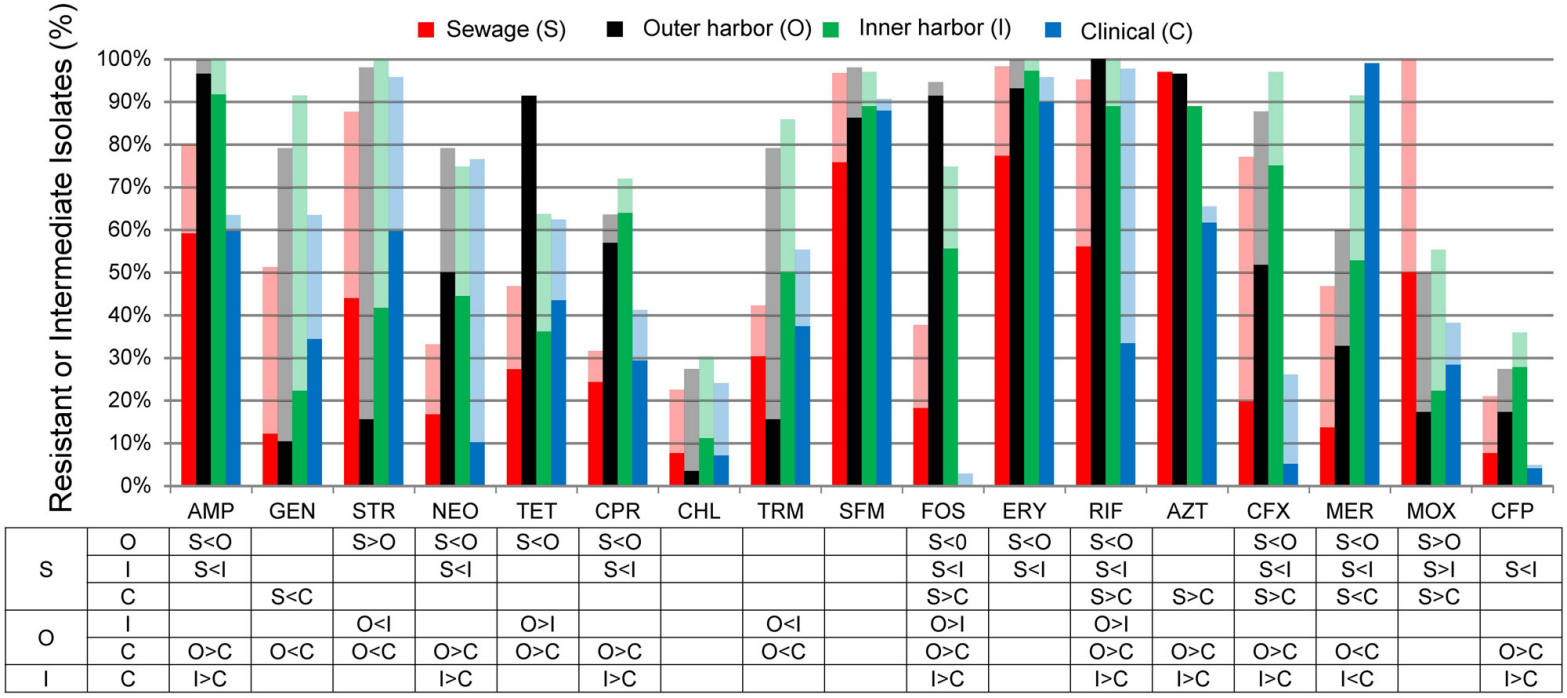
**Percentage of *Escherichia coli* isolates from sewage (S, *n* = 66), outer harbor (O, *n* = 58), inner harbor (I, *n* = 36), and clinical setting (C, *n* = 99) indicating resistance (solid) or intermediate resistance (transparent) to 17 different antibiotics.** Table indicates significant difference in antibiotic resistance (not including intermediate resistance) between locations (*p* < 0.01) by proportional *Z* test.

**FIGURE 2:**
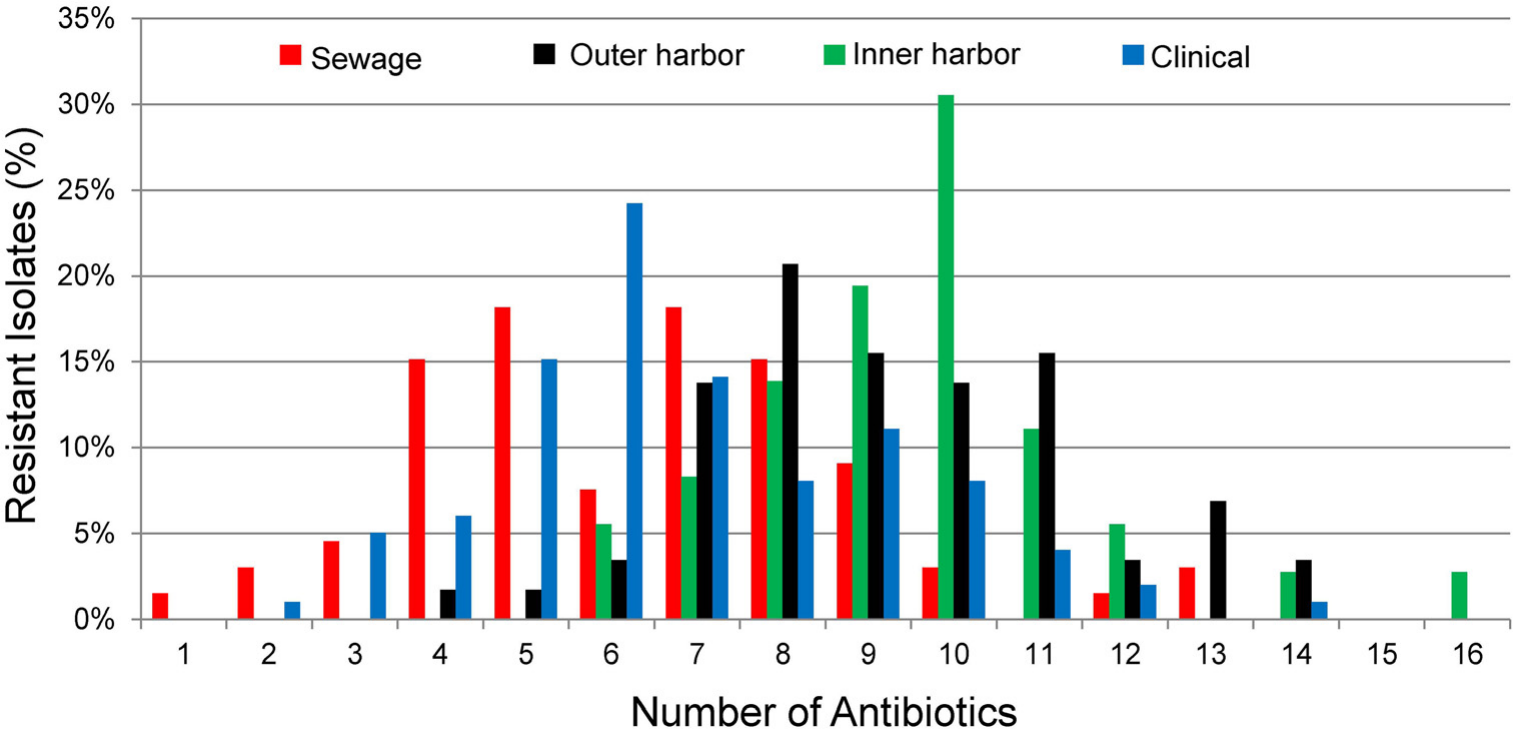
**Percentage of *E. coli* isolates from sewage (*n* = 66), outer harbor (*n* = 58), inner harbor (*n* = 36), and clinical setting (*n* = 99) showing number of antibiotic resistances (Resistant-level)**.

The antibiotic resistance patterns of the *E. coli* isolates to 17 antibiotics are shown in **Figure 1**. Duplicate isolates were determined and eliminated based on phenotypic and genotypic results; however, no duplication was observed. Resistant-level phenotype, growth at the highest concentrations of antibiotic within the phenotypic assay, was detected for all antibiotics tested. All isolates (*n* = 259) showed a resistant-level phenotype for at least one antibiotic and non-sensitive level phenotype, growth at any concentration of antibiotic tested within the phenotypic assay, for at least six antibiotics tested (**Figure 2**; Supplementary Figure S2). All *E. coli* isolates were non-sensitive to at least one agent in three or more antibiotic categories as defined for multi-drug resistant (MDR) bacteria in Magiorakos et al. (2012). Three isolates, two from the inner harbor and one from the outer harbor were non-sensitive (intermediate or resistant level of phenotypic antibiotic resistance) to all 17 antibiotics tested. These three strains resistant to all antibiotics tested are potential extensively drug-resistant (XDR) bacteria showing non-sensitivity to 11 of the required 15 out of 17 antibiotic categories (Magiorakos et al., 2012). Antibiotic resistance was most prevalent for erythromycin, sulfamethoxazole, aztreonam, and ampicillin occurring in 72–88% of all isolates. The least prevalent antibiotic resistance in all isolates was for chloramphenicol, observed in less than 7%.

*E. coli* isolates collected in the inner or outer harbor of Milwaukee demonstrated significantly greater frequencies of resistances to eight antibiotics compared to the *E. coli* isolates collected from human derived sewage and of clinical concern (*p* < 0.01; **Figure 1**). These eight antibiotics included ampicillin, neomycin, tetracycline, ciprofloxacin, fosfomycin, rifampicin, cefuroxime, and cefepime. The isolates from the urban waterways additionally showed significantly greater frequencies of resistance to erythromycin compared to isolates from human derived sewage (*p* < 0.01) and aztreonam compared to clinical isolates (*p* < 0.01). Of the antibiotics tested, the isolates in the outer harbor collected near the WWTP eﬄuent showed significantly greater frequencies of tetracycline, fosfomycin, and rifampicin resistance compared to the inner harbor isolates (*p* < 0.01).

Since all *E. coli* isolates tested were determined to be MDR bacteria, the distribution of the number of antibiotics the isolates were resistant was used to explore the differences between the urban waterway and human derived isolates. The number of antibiotic resistances in the *E. coli* isolates was greater from the inner harbor (median = 9, range: 6–16 antibiotics) and outer harbor (median = 10, range: 4–14) of the urban waterways compared to the clinical isolates (median = 6, range: 2–12) and isolates from human derived sewage (median = 6.5, range: 1–13; **Figure 2**, *p* < 0.001). (The number of antibiotic resistance in the *E. coli* isolates demonstrating non-sensitive phenotypes similar to determine MDR are in Supplementary Figure S2.) There was no significant difference in the number of antibiotics an isolate was resistant between inner and outer harbor isolates (*p* = 0.52) nor human derived sewage and clinical isolates (*p* = 0.17).

**FIGURE 3:**
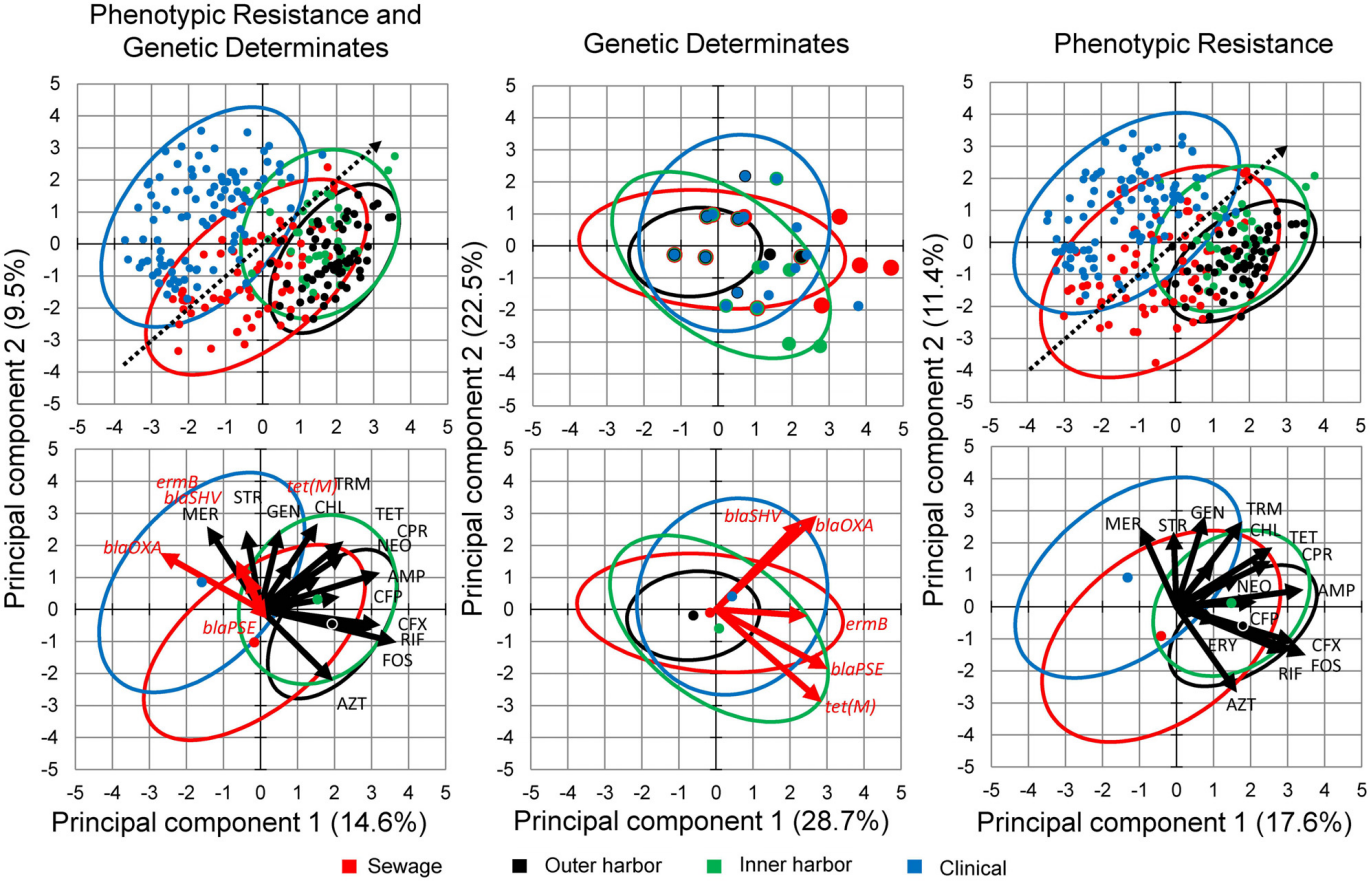
**Principal Component Analysis (PCA) biplots of *E. coli* isolates in terms of their phenotypic antibiotic resistance profiles and/or their genotype based on the presence of antibiotic resistance genes.** Separate PCA were performed on data containing both the phenotypic and genotypic profiles **(Left)** and the genotypic **(Middle)** and phenotypic **(Right)** profiles separately. The green points represent the *E. coli* isolates from the inner Milwaukee Harbor, black points from the outer harbor, red points from human derived sewage, and blue from clinical isolates. Points in the upper panels are individual isolates and the points in the bottom panels represent the group center (average of individuals). Dotted line in the upper panels shows the gradient of number of resistance (multiple antibiotic resistances) in the isolates. The ellipses represent a 95% confidence interval of the respective points in the same color. In the right panel: Black arrows represent antibiotics (see Materials and Methods for abbreviations) utilized in the phenotypic antibiotic resistance screening. Erythromycin (ERY), Sulfamethoxazole (SFM), and Moxifloxacin (MOX) respective arrows may not be perceptible due to little influence in the ordination. Red arrows represent antibiotic resistance genes screened by PCR.

Principal component analysis of the combined genotype and phenotypic resistance profiles for each isolate was used to explore the difference in the environmental resistome from which the *E. coli* isolates were derived (**Figure 3**). (**Figure 3** contains genotype and phenotype in separate PCA analysis as well.) PCA analysis demonstrated a gradient related to number of antibiotics the *E. coli* isolates were resistant to (**Figure 3**, dotted arrow). Permutational MANOVA analysis showed significant differences between isolates from the different sources (*p* < 0.001) and follow up pairwise comparison showed significant differences between all sources (*p* < 0.01) with the exception of the human derived sources (clinical and human derived sewage, *p* = 0.74).

Isolates collected from human derived sewage were positively correlated with moxifloxacin resistance (*p* < 0.001). The gene *bla*_OXA_ encoding a β-lactamase showed significant (*p* < 0.001) positive correlation with the clinical isolates. The vectors related to the phenotypic antibiotic resistance for meropenem, a mono-β-lactam (*p* < 0.001) were also significantly correlated with the clinical isolates. The inner harbor isolates positively correlated with antibiotic resistance for fosfomycin, UDP-*N*-acetylglucosamine enolpyruvyl transferase inhibitor (*p* < 0.01), cefuroxime, a second generation cephalosporin (*p* < 0.001), and the presence of the *tet*(M) (*p* < 0.01) gene encoding tetracycline resistance. The outer harbor isolates were positively correlated with resistance to tetracycline (*p* < 0.001), fosfomycin (*p* < 0.001) and cefuroxime (second generation cephalosporin; *p* < 0.01).

The PCA analysis suggested putative incidences of co-occurrences of resistances across sources. The co-occurrences of non-sensitive phenotypes (resistance and intermediate levels) for combinations of antibiotics were recurrent. **Table 1** shows the co-occurrences of non-sensitive phenotypes to 3–5 antibiotics demonstrated within 20% or more of the isolates from a location. The majority of co-occurrences of resistances were identified within the urban waterways. The presence of the co-occurrences in the urban waterways is expected due to the high incidences of resistance in the isolates (**Figure 1**) and the number of resistance within the isolates (**Figure 2**). The co-occurrences of ampicillin, fosfomycin, rifampicin, and cefuroxime resistance were of the greatest abundance in urban waterway isolates compared to human derived sewage and clinical *E. coli* isolates. The co-occurrences of neomycin, sulfamethoxazole, erythromycin, and meropenem resistance were not significantly different between the urban waterways and clinical isolates indicating a potentially common resistance mechanism between the environmental and the clinical *E. coli* isolates.

**Table 1:** The percentage of *Escherichia coli* isolates from the urban waterways of the inner and outer Milwaukee Harbor and of human derived sources (sewage and clinical) with co-occurrence of antibiotic resistance.

Co-occurrence of antibiotic resistance^1^	Inner harbor (36)^2^	Outer harbor (58)	Sewage (66)	Clinical (99)	Significance (*p* < 0.01)^3^
GEN, STR, TRM, AZT, MER	58.3 (21)	37.9 (22)	12.1 (8)	22.2 (22)	O > S, I > S, I > C
AMP, FOS, RIF, CFX	75.0 (27)	82.8 (48)	25.8 (17)	2.0 (2)	O > S, I > S, O > C, I > C
NEO, SFM, ERY, MER	66.7 (24)	55.0 (29)	18.2 (12)	69.7 (69)	O > S, I > S, C > S, O > C
GEN, STR, CHL, MER	27.8 (10)	8.6 (5)	7.6 (5)	14.1 (14)	I > S, I > O
GEN, TET, SFM	58.3 (21)	70.7 (41)	28.8 (19)	39.4 (39)	O > S, I > S, O > C
TET, TRM, SFM	38.9 (14)	36.2 (21)	28.8 (19)	24.2 (24)	NS
STR, ERY, RIF	72.2 (26)	75.9 (44)	30.3 (20)	54.5 (54)	O > S, I > S, C > S, O > C

^1^Criteria for inclusion was non-sensitive resistance (resistance or intermediate resistance level) for three or more antibiotics and presence in greater than 20% in one or more locations.

^2^Reported in percentage of isolates from location and number of isolates in parenthesis.

^3^Statistical significance was determined between locations by proportional *Z* test.

In summary, a higher incidence of antibiotic resistance, prevalence of resistance to a greater number of antibiotics and a greater incidence of co-occurrences of resistance was identified within the urban waterway (inner and outer Milwaukee Harbor) *E. coli* isolates compared to the human derived isolates (sewage and clinical isolates) from Milwaukee.

### Detection of Antibiotic Resistance Genes

**FIGURE 4:**
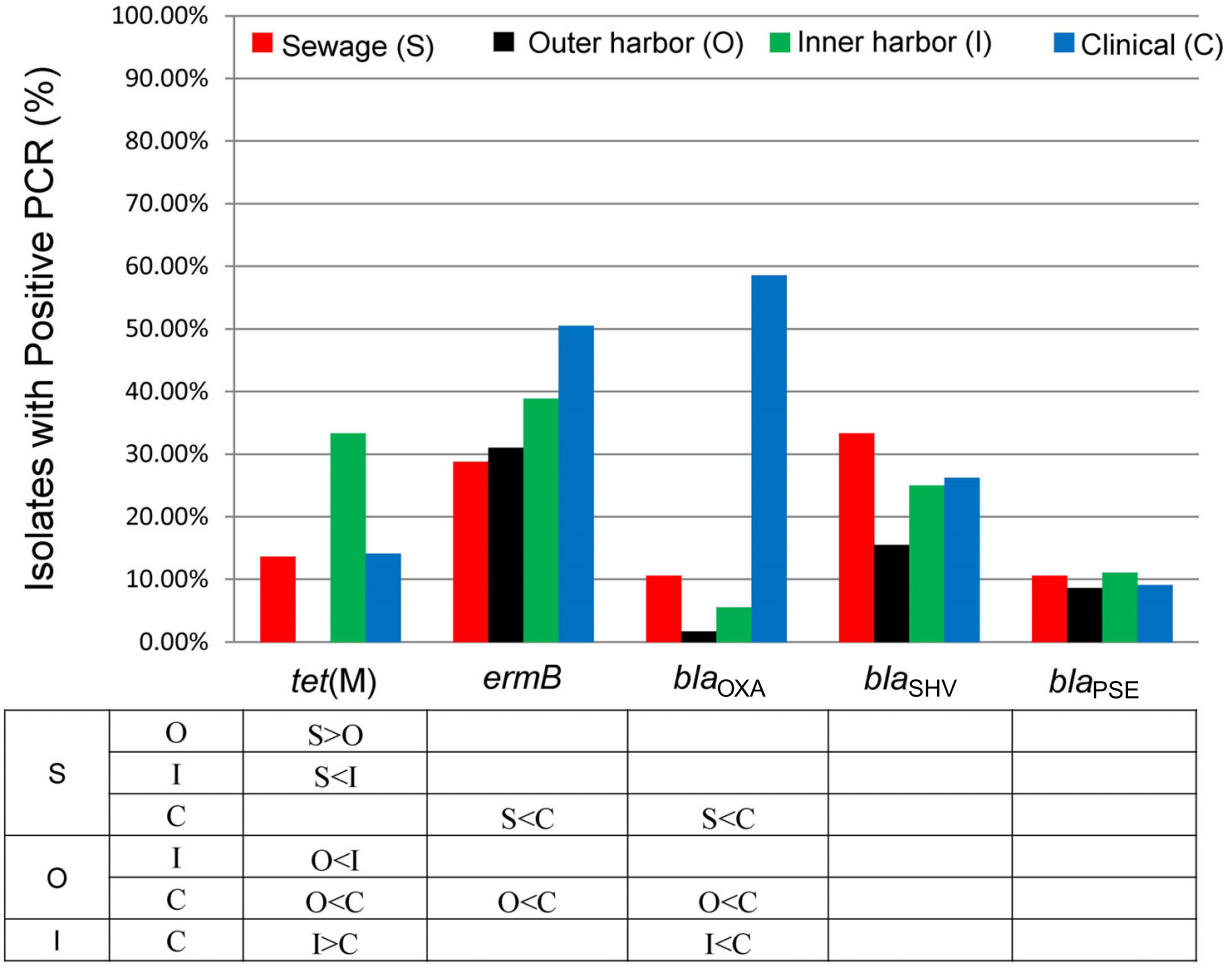
**Percentage of *E. coli* isolates from sewage (*n* = 66), outer harbor (*n* = 58), inner harbor (*n* = 36), and clinical setting (*n* = 99) harboring antibiotic resistance genes.** Table indicates significant difference in antibiotic resistance (not including intermediate resistance) between locations (*p* < 0.01) by proportional *Z* test.

The presences of five antibiotic resistance genes: *ermB* (macrolide resistance), *tet*(M), and β-lactamases (*bla*_OXA_, *bla*_SHV_, and *bla*_PSE_) were screened for in the *E. coli* isolates (**Figure 4**). More than 65% of all the *E. coli* isolates (*n* = 259) harbored at least one of the five resistance genes chosen for analysis. The presence of *ermB* was the most prevalent represented in 38% of the isolates. The β-lactamase encoding genes *bla*_OXA_ and *bla*_SHV_ were present in 26 and 25% of all strains, respectively. The resistance gene *bla*_PSE_ and *tet*(M) were the least prevalent at 10 and 14%, respectively. The clinical *E. coli* isolates showed significantly greater frequencies of the *bla*_OXA_ compared to the urban waterway isolates of the inner and outer harbor and the human derived sewage (**Figure 4**). The greater incidence of resistance to meropenem in the clinical *E. coli* isolates can be partially (58.2%) explained by the greater frequency of the *bla*_OXA_ gene in the isolates compared to less than 10% in the meropenem resistant *E. coli* isolates from urban waterways and human derived sewage (**Table 2**). The presence of any β-lactamase genes could explain 73% of the ampicillin resistant clinical *E. coli* isolates compared to only 21–40% of the ampicillin resistant *E. coli* isolates from the urban waterways and human derived sewage.

**Table 2:** Percentage of antibiotic resistance in *E. coli* isolates from different locations explained by the presence of a gene able to confer the resistance.

Antibiotic	Gene^1^	Inner harbor^2^	Outer harbor	Sewage	Clinical
Penicillins (AMP)	SHV, PSE, OXA	36.1 (13/36)	20.7 (12/58)	39.6 (21/53)	73.0 (46/63)
Second generation Cephalosporin (CFX)	SHV, PSE	34.3 (12/35)	23.5 (12/51)	33.3 (17/51)	34.6 (9/26)
Fourth generation Cephalosporin (CFP)	SHV, PSE	53.9 (7/13)	12.5 (2/16)	14.3 (2/14)	60.0 (3/5)
Carbapenems (MER)	OXA	3.03 (1/33)	2.9 (1/35)	9.7 (3/31)	58.2 (57/98)
Monobactam (AZT)	SHV, PSE	34.4 (11/32)	19.6 (11/56)	32.8 (21/64)	35.4 (23/65)
Tetracycline (TET)	*tet*(M)	39.1 (9/23)	0.0 (0/53)	16.1 (5/31)	8.1 (5/62)
Macrolide (ERY)	*ermB*	38.9 (14/36)	31.0 (18/58)	29.2 (19/65)	51.6 (49/95)

^1^Genes encoding β-lactamases are abbreviated to their types (i.e., *bla*_*SHV*_ as SHV).

^2^Percentages represent the number of strains from a location that have a non-sensitive resistance (resistance or intermediate resistance level) for an antibiotic and the presence of at least one gene listed in the table. In parenthesis: (number of isolates with gene/total with non-sensitive resistance).

The *E. coli* isolates of the inner harbor demonstrated greater frequencies of the *tet*(M) gene compared to the isolates of the outer harbor and derived from human sources (clinical and human derived sewage isolates). The presence of the *tet*(M) gene explained greater than 38.9% of the tetracycline resistant isolates detected in the inner harbor and less than 16.1% in the other tetracycline isolates from other sources.

The *ermB* gene was present in significantly greater frequency in clinical *E. coli* isolates than the *E. coli* isolates from the outer harbor and human derived sewage. The presence of *ermB* may explain 52% of the erythromycin resistance observed in the clinical isolates, while less than 40% of erythromycin resistance could be explained in the urban waterways and human derived sewage isolates.

The few genetic determinants detected in this study had little to no effect on the PCA analysis in the presence of the phenotypic data (**Figure 3**). Utilizing only the genetic determinations in PCA analysis and MANOVA did not show any significant difference between sources of *E. coli* isolates.

While the genetic determinates were able to explain some of the high incidences of resistance within the clinical *E. coli* isolates, direct phenotypic determination of antibiotic resistance showed greater incidences of antibiotic resistance and greater number of resistance within the *E. coli* isolates from urban waterways.

## Discussion

The emergence and dispersion of antibiotic resistance has reduced the susceptibility of pathogens to antibiotics in medical treatment. A detailed examination of the origin and role of antibiotic resistance in natural environments is necessary to understand the evolution and dissemination of antibiotic resistance genes in pathogens (Allen et al., 2010). The urban rivers merging into Milwaukee Harbor and ultimately Lake Michigan allowed for a representative assessment of the distribution of antibiotic resistance within these urban-influenced environments.

Prevalence of resistance to erythromycin, sulfamethoxazole, aztreonam, and ampicillin was detected in all studied sources. Occurrence of resistance and resistant determinates related to β-lactams, sulfonamides, and macrolides are not uncommon in urban settings (Hu et al., 2008; Munir et al., 2011; Rodríguez-Mozaz et al., 2014; Yang et al., 2014). The detection of macrolides and sulfonamides within the influent and eﬄuent of a municipal wastewater treatment plant servicing the greater Milwaukee, WI area (Blair et al., 2013b) may indicate high historical usage of these antibiotics which may have led to establishment of resistant *E. coli* within the bacterial communities observed in this study. Similar levels of antibiotic resistance in the sediment of the urban waterways of Milwaukee compared to raw sewage have been detected previously (Salmore et al., 2006). The low prevalence of chloramphenicol resistance in this study is consistent with published data within the U. S. (Johnson et al., 2013) and supports the idea that this old-generation antibiotic has the potential to address the current need for new antibiotics despite the potential of negative side-effects (Falagas et al., 2008).

The high rate of MDR classification within the isolates used in this study may be due to choice of older individual antibiotics within the antibiotic classes as representatives to distinguish individual isolates based on phenotypic resistance. Previous studies have indicated lower percentages of MDR compared to this study (Baquero et al., 2008; Figueira et al., 2011; Ham et al., 2012). However, these studies used more recently deployed or fewer antibiotics compared to this study making it less likely to establish MDR.

The *E. coli* isolates from urban waterways demonstrated greater incidence of resistance to single antibiotics and multiple antibiotic resistance within a single isolate compared to human derived sources (sewage and clinical isolates). The observation of greater incidence of antibiotic resistance in *E. coli* from natural waters compared to a possible sources of fecal contamination is consistent with the observations of Alves et al. (2014), which showed a higher incidence of multi-resistant *E. coli* in coastal waters of an uninhabited island than the source of fecal contamination by seagull feces and human sewage from tourist. The higher level of resistance within the urban waterway *E. coli* isolates may be explained by the historically high abundance of antibiotics, in particular of macrolides, fluoroquinolones, and sulfonamides, quantified within the water and sediments of the Milwaukee Harbor (Blair et al., 2013a). These antibiotics may have synergistic selective effect on mechanisms of antibiotic resistance leading to increased selective pressure for antibiotic resistance within the microbial population (Davies et al., 2006; Andersson and Hughes, 2014). In other urban waterway environments receiving treated wastewaters a higher incidence and chronic presence of antibiotics and antibiotic resistance genes were detected downstream of released eﬄuent (LaPara et al., 2011; Munir et al., 2011; Korzeniewska et al., 2013; Alves et al., 2014; Czekalski et al., 2014; Rodríguez-Mozaz et al., 2014).

The greater prevalence of the antibiotic resistance co-occurrences in urban waterways may point to a mobile resistome within the microbial population of urban waterway sediment. Previous studies tend to use a strict criteria for multiple resistance shared between isolates when exploring phenotypic similarities between locations and sources (Hu et al., 2008; Alves et al., 2014). For example, isolates showing resistance to four antibiotics would be grouped, while an isolate resistant to the same four antibiotics with additional resistances would be excluded from the grouping. This restriction may led to misinterpretation in the prevalence of specific multiple resistances shared between isolates. The prevalence of resistance to specific antibiotics or antibiotic classes within a microbial population may be explained by the presence of mobile genetic elements within the population encoding that antibiotic resistance (Thavasi et al., 2007). The existence of plasmids bearing extended spectrum β-lactamase (ESBL) and fosfomycin resistance determinants that can spread effectively in *Enterobacteriaceae* have been discovered and are of great clinical concern (Zhao et al., 2015). A high prevalence of co-occurrence of resistance to fosfomycin, ampicillin, cefuroxime, and rifampicin and other patterns within the urban waterways of Milwaukee may be explained by the existence of mobile genetic elements encoding the resistances for these antibiotics within the microbial community. The detection of the antibiotic resistance genes *ermB*, *tet*(M), *bla*_OXA_, *bla*_SHV_, and *bla*_PSE_ (**Figure 1**) which are associated with natural plasmids (Brisson-Noel et al., 1988; Carattoli, 2013) and transposons (Bryan et al., 2004) within the *E. coli* isolates of the urban waterways supports the possible presence of these type of mobile genetic elements.

The greater incidence of resistance observed in the urban waterways (**Figure 1**) was not fully explained by the presence of the resistance genes tested (**Table 2**). The genes *ermB* and *bla*_OXA_ were present in a greater percentage of isolates in the clinical *E. coli* isolates. While the transfer of the resistance gene *ermB* from Gram-positive cocci to *E. coli* (Brisson-Noel et al., 1988) was previously detected, very few studies attempt to identify and quantify this gene in non-Gram-positives despite the ability to confer resistance (Goetting-Minesky and Fenno, 2010). The *ermB* gene has also been identified in *Bacteroides* (Shoemaker et al., 2001) and *Campylobacter coli* (Qin et al., 2013). To our knowledge, this study is the first to attempt to quantify the presence of *ermB* in *E. coli* from environmental waters. The emergence of *bla*_OXA_ carbapenemase producing *Enterobacteriaceae* is difficult to detect because of their relative low MICs (Seiffert et al., 2014). The detection of *bla*_OXA_ is consistent with expectations in the environment (Ojer-Usoz et al., 2014) and clinical isolates (Kaftandzieva et al., 2014). One possible explanation for the lower presence of the genes tested in *E. coli* from the urban waterways to that of the human derived sources may be related to survival in non-human environments. The selective pressure on the *E. coli* in the sediment of the urban waterways from various physical conditions including nutrient availability, temperature, and predation (Beversdorf et al., 2007) and chemicals including antibiotics, personal care products, and metals may select for phenotypes that have co-selection for mechanisms of antibiotic resistance other than the ones encoded by the studied genes (Graham et al., 2010). Co-selection can also be related to cross-resistance, such as multi-drug resistance pumps where the primary roles is thought to provide tolerance to toxic compounds in the environment (Poole, 2005; Martinez et al., 2009; Allen et al., 2010). An additional explanation of the higher incidence of antibiotic resistance within the urban waterways is the selection of antibiotic resistance within the bacteria prior to entering the environment. The sources of the antibiotic resistant *E. coli* in this study can include the WWTP (Korzeniewska et al., 2013) as the major contaminating source was previously determined to be human in the study area (Newton et al., 2013). Determining the resistances from the contaminating bacterial sources may yield information to determine if the resistances observed in this study are due to depositing of resistance or selection of resistance.

## Conclusion

This study demonstrates urban waterway sediment as a relevant reservoir of *E. coli* strains containing multiple resistances to antibiotics and antibiotic resistance genes. The presence of known antibiotic resistance genes harbored on mobile genetic elements and a greater level of resistance compared to the local human derived *E. coli* suggests that sediment of the urban waterways may harbor an extensive mobile resistome. Further work is needed to explore the presence of a putative mobile resistome, its contribution to the reservoir of antibiotic resistance, and the potential of transfer to human commensal bacteria and pathogens.

## Author Contributions

AK and KH conceived and designed the experiments. AK, MD, and NA performed the experiments. NL, RN, and KH contributed materials, reagents, strains, and tools for analysis. The manuscript was prepared by AK, RN, and KH.

## Conflict of Interest Statement

The authors declare that the research was conducted in the absence of any commercial or financial relationships that could be construed as a potential conflict of interest.
