# Supplementary material for: Detection of multi-drug resistant Escherichia coli in the urban waterways of Milwaukee, WI
Source: Front Microbiol. 2015 Apr 29;6:336. doi: 10.3389/fmicb.2015.00336 (PMC4413672; doi:10.3389/fmicb.2015.00336)
Supplement: Supplementary file 1 [file Presentation_1.PDF]

SUPPLEMENTAL DATA

Detection of multi-drug resistant *Escherichia coli* in the urban waterways of Milwaukee, WI

Anthony D. Kappell[1], Maxwell S. DeNies[1], Neha H Ahuja[1], Nathan A. Ledeboer[2,3], Ryan J. Newton[4], and Krassimira R. Hristova[1*]

[1]Department of Biological Sciences, Marquette University, Milwaukee, WI, USA
[2]Department of Pathology, Medical College of Wisconsin, Milwaukee, WI, USA
[3]Dynacare Laboratories, Milwaukee, WI, USA
[4]School of Freshwater Sciences, Great Lakes WATER Institute, University of Wisconsin-Milwaukee, Milwaukee, WI, USA

Correspondence:
Dr. Krassimira R. Hristova
Marquette University
Department of Biological Sciences
P.O. Box 1881
Milwaukee, WI 53201-1881, USA
krassimira.hristova@marquette.edu

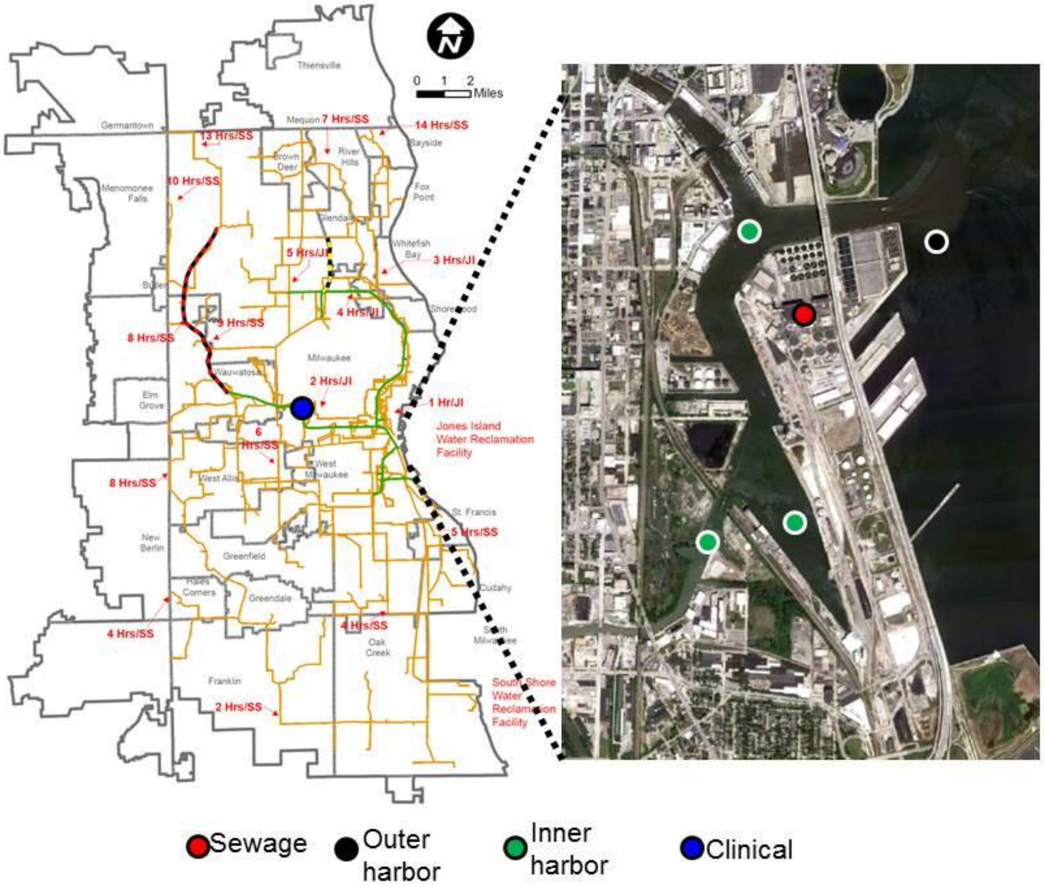

Figure S1. Sampling site map and wastewater travel times for the region of Milwaukee, WI. Map on left is of the wastewater travel times of the Milwaukee Metropolitan Sewage District to the Jones Island WWTP and the South Shore WWTP. The maximum travel time is 5 hours for the Jones Island WWTP serviced area. The right map is adapted from Google Maps. *E. coli* from human derived sewage were isolated from influent wastewater to the Jones Island WWTP represented in red. The locations of sediment samples utilized for the *E. coli* isolates from the inner harbor are in green. In black is the location of the sediment sample for the outer harbor *E. coli* isolates. The clinical isolates were from Dynacare clinical microbiology laboratory near a hospital complex represented in blue. The Dynacare and nearby area's waste is serviced by the Jones Island WWTP with a travel time of approximately 2 hours.

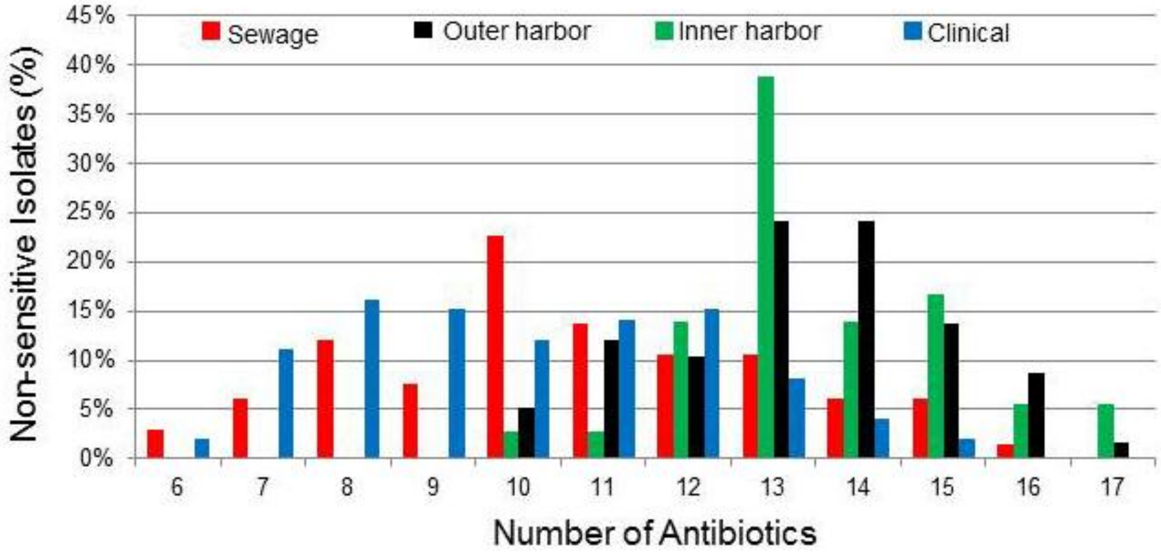

Figure S2. Percentage of *E. coli* isolates from sewage (n =66), outer harbor (n=58), inner harbor (n=36), and clinical setting (n=99) showing number of non-sensitive antibiotic resistances (resistance and intermediate resistance).

Table S1. PCR Primer Sequences

Target	Primer	Sequence (5'-3')	Annealing Condition	Product Size	Related resistances	Reference
tet(M)	TetM-F<br>TetM-R	GGTTTCTCTTGGATACTTAAATCAATCR<br>CCAACCATAYAATCCTTGTTCRC	60	87	Tetracycline	(Graham et al. 2010)
ermB	ErmB-F<br>ErmB-R	AAAACTTACCCGCCATACCA<br>TTTGGCGTGTTTCATTGCTT	60	137	Lincosamide, Marcolide, Streptogramin B	(Graham et al. 2010)
bla_{OXA}	OXA1B14<br>blaOXA1-R	CACTTACAGGAAACTTGGGGTCG<br>AGTGTGTTTAGAATGGTGATC	55	78	Penicillins, Carbapenems	(Graham et al. 2010)
bla_{SHV}	RTblaSHVF<br>RTblaSHVR	CGCTTTCCCATGATGAGCACCTTT<br>TCCTGCTGGCGATAGTGGATCTTT	64	94	Penicillins, Cephalosporins, Monobactams	(Marti et al. 2013)
bla_{PSE}	PSE-F<br>PSE-R	ACCGTATTGAGCCTGATTTA<br>ATTGAAGCCTGTGTTTGAGC	55	321	Penicillins, Cephalosporins, Monobactams	(Bert et al. 2002)

Bert, F., C. Branger and N. Lambert-Zechovsky (2002). "Identification of PSE and OXA β-lactamase genes in *Pseudomonas aeruginosa* using PCR–restriction fragment length polymorphism." Journal of Antimicrobial Chemotherapy **50**(1): 11-18.

Graham, D. W., S. Olivares-Rieumont, C. W. Knapp, L. Lima, D. Werner and E. Bowen (2010). "Antibiotic resistance gene abundances associated with waste discharges to the Almendares River near Havana, Cuba." Environmental science & technology **45**(2): 418-424.

Marti, E., J. Jofre and J. L. Balcazar (2013). "Prevalence of antibiotic resistance genes and bacterial community composition in a river influenced by a wastewater treatment plant." PloS one **8**(10): e78906.

Table S2. Summary of the number of isolates sequenced to confirm PCR amplification

Target	Inner Harbor	Outer Harbor	Sewage	Clinical
ermB	9 (14)[1]	10 (18)	11 (19)	11 (50)
tet(M)	9 (12)	0 (0)	8 (9)	11 (14)
OXA	2 (2)	1 (1)	5 (7)	17 (58)
SHV	4 (9)	6 (9)	12 (22)	13 (26)
PSE	3 (5)	3 (5)	6 (7)	5 (9)

[1] Number of isolates sequenced for confirmation and in parenthesis number of isolates positive by PCR. All PCR results sequenced were confirmed for target sequence.
